# Virus matrix interference on assessment of virucidal activity of high-touch surfaces designed to prevent hospital-acquired infections

**DOI:** 10.1186/s13756-021-01001-x

**Published:** 2021-09-10

**Authors:** Sadru-Dean Walji, Mark R. Bruder, Marc G. Aucoin

**Affiliations:** Department of Chemical Engineering, University of Waterloo, 200 University Ave West, Waterloo, ON N2L 3G1 Canada

**Keywords:** Hospital-acquired infection, Healthcare-associated infection, HAI, Nosocomial infection, Virucide, Copper, Copper alloy, Self-sterilizing

## Abstract

**Objectives/purpose:**

High-touch surfaces are a critical reservoir in the spread of nosocomial infections. Although disinfection and infection control protocols are well developed, they lack the ability to passively reduce the pathogenic load of high-touch surfaces. Copper and its alloys have been suggested as a surface that exhibit continuous biocidal effects. Antimicrobial studies on these surfaces are prevalent, while virucidal studies are not as well explored. The goal of this study was to first determine the virucidal activity of a copper–nickel–zinc alloy and to then examine the effect of soiling and virus preparation on virucidal activity.

**Methods:**

A baculovirus vector was used as an easily quantifiable model of an infectious enveloped animal cell virus. Droplets containing virus were deposited on surfaces and allowed to stay wet using humidity control or were dried onto the surface. Virus was then recovered from the surface and assayed for infectivity. To examine how the composition of the droplet affected the survival of the virus, 3 different soiling conditions were tested. The first two were recommended by the United States Environmental Protection Agency and the third consisted of cell debris resulting from virus amplification.

**Results:**

A copper–nickel–zinc alloy was shown to have strong virucidal effects for an enveloped virus. Copper, nickel, and zinc ions were all shown to leach from the alloy surface and are the likely cause of virucidal activity by this surface. Virucidal activity was achieved under moderate soiling but lost under high soiling generated by routine virus amplification procedures. The surface was able to repeatably inactivate dried virus droplets under moderate soiling conditions, but unable to do so for virus droplets kept wet using high humidity.

**Conclusion:**

Ion leaching was associated with virucidal activity in both wet and dried virus conditions. Soiling protected the virus by quenching metal ions, and not by inhibiting leaching. The composition of the solution containing virus plays a critical role in evaluating the virucidal activity of surfaces and surface coatings.

## Introduction

Nosocomial infections are a large burden on heath-care systems, with the American Center for Disease Control (CDC) estimating one in every 25 patients acquiring one. In 2009, a CDC summary indicated that a conservative cost-estimate of preventable nosocomial infections ($5.7–$6.8 billion) was comparable to the cost for treating stroke, complications from diabetes, or chronic obstructive pulmonary disease [1]. These types of infections are known to occur through medical devices or environmental transmission, and strategies have been developed to reduce them.

The key to environmental transmission is an intermediary reservoir, and transmission barriers can be developed to target these reservoirs. Currently, there are two major avenues: increased handwashing compliance, and routine cleaning/disinfection protocols. The first avenue is strongly targeted at healthcare professionals, and simply seeks to increase hand-washing compliance through monitoring and awareness [2]. While this does address a key transmission route for nosocomial infections, compliance is inconsistent and requires oversight [3]. The second avenue, disinfection, and cleaning, also faces consistency problems, with inconsistency in methods utilized by custodians [4]. A newer avenue is to design surfaces that are biocidal against key pathogens. This method may add initial costs, but it does allow for greater consistency and has potential to decrease costs incurred in treatment of nosocomial infections. Development of these surfaces does not endeavour to displace established cleaning and disinfection procedures, but to supplement them and create a comprehensive defence against environmental transmission.

Copper-based surfaces have antimicrobial properties, something known for several centuries. However, formal recognition was only obtained in 2008, when the United States Environmental Protection Agency (EPA) designated copper as an antimicrobial surface. Initial studies have shown copper to be an effective antimicrobial, even showing complete reduction of copper-tolerant bacterial species [5]. Unfortunately, research has shown that pure copper performs better than most common copper alloys [6]. However, using pure copper is impractical as it is soft, malleable, and tarnishes. It would be advantageous to develop an alloy coating robust against the high-frequency touching these surfaces sustain.

While work has been done to show the antibacterial properties of copper, comparatively little has been done to show virucidal effects of such surfaces. Although, bacteria and fungal spores are considered the primary threat for nosocomial infections, viral infections make up an estimated 60% of all infections [7]. Further, recent work shows that viral nosocomial infections are underestimated [8] and under-reported [9]. Virus, like bacteria, can be transferred to multiple surfaces, with one study finding a soiled hand could spread norovirus to multiple subsequently touched surfaces [10]. This spread of virus adds to the existing need to replace high-touch stainless steel with self-sterilizing surfaces.

A problem with evaluating virucidal properties of these alloys is that no purpose-made standard tests are available. This leads to difficulty in comparisons of efficacy to established disinfectants. The EPA is currently developing a standard test that evaluates antimicrobial activity under actual use conditions [11, 12], but does not test the virucidal properties of the surface. Alternatively, existing protocols for disinfectant sprays can be adapted to investigate the virucidal properties of self-sterilizing surfaces. However, the danger of using these parallel protocols was demonstrated by Hodek et al. [13], where virucidal silver was effective in the standard’s test conditions, but not in use-conditions. The lack of clear guidance has led to a variety of methods being tested in literature [14].

A major source of variability in protocols is the soiling agent. Organic debris generated as part of the infection when the virus lyses the host and is composed of intracellular nucleic acids and proteins. In this context, the organic debris is termed a soiling agent. Soiling is a known interfering factor of disinfectant tests [15], and is included in several parallel standard tests. However, the quantity and type of soil varies between standards organizations and intended use of the disinfectant.

The goal of this study was to first determine the virucidal activity of a copper–nickel–zinc alloy. Then, the effect of soiling and virus preparation were investigated. After which repeatable inactivation by the surface was explored. Finally, the alloy’s constituent metals were tested for virucidal activity as ionic species with and without soiling. Using a BSL-1 *Autographa californica* multiple nucleopolyhedrovirus virus vector (baculovirus vector), we investigated the above parameters either by allowing the droplet to stay wet using humidity control (standard test conditions), or by drying a virus droplet (actual conditions) onto the alloy and stainless steel.

## Materials and methods

### Cell culture and virus production

*Spodoptera frugiperda* cells (Sf-9, ATCC CRL-1711™), able to support the replication of baculovirus vectors, were grown in serum-free SF-900 III (Gibco BRL, Life Technologies, Burlington, Ont., Canada). Cells were routinely maintained in 125 mL glass shake flasks (Corning GlassWorks, Corning, NY) with a working volume of 30 mL at 27 °C and agitated at 130 rpm. Cells were sub-cultured twice per week to maintain the cell density between 0.5 and 5 × 10^6^ cells/mL. Cell densities were assessed using a Countess^TM^II FL Automated Cell Counter (Invitrogen, FisherSci, Ont., Canada). Cell viability was determined via the trypan blue exclusion method.

Baculovirus (*Autographa californica* multiple nucleopolyhedrovirus) was engineered to express the fluorescent protein mKOk under the late and strong viral p10 promoter using the Bac-to-Bac™ Baculovirus Expression System (Fisher Scientific, Ontario, Canada). Briefly, the mKOk gene was amplified from plasmid Chicken Mermaid S188 (Addgene #53617) [16] and inserted into the pFastbac1 transfer plasmid that was modified to include the p10 promoter and 3’ untranslated region (UTR) in place of the polh/SV40 3’UTR. A passage 2 working stock of the virus was made by infecting 2 × 10^6^ cells/mL of exponentially growing Sf-9 cells with recombinant virus at a MOI of 0.01. Cell viability was measured daily until it was between 80 and 60% (72–96 h post-infection), after which virus supernatant was harvested, purified by low-speed centrifugation (800×*g*, 10 min) and filtration through a 0.2 µm filter. Purified virus was stored at 4 °C until use.

The enveloped baculovirus was selected for its safe handling and ease of manipulation. Using genetic manipulation to express a fluorescent protein allows a more consistent and accurate judgment over cytopathic effects when scoring end-point dilution assays. Further, red fluorescent protein was selected to avoid the auto-fluorescence of growth media when exciting green fluorescent protein. Auto-fluorescence can mask fluorescence from infected cells, thereby lowering accuracy of the end-point dilution assay.

### Test surface preparation

Aereus Technologies provided copper–nickel–zinc alloy (UNS C75200) coated stainless steel and stainless steel (UNS S30400) coupons (1 × 1 × 1/16 in). Alloy-coated coupons were created using a liquid-spray application process and had a semi-polished finish on both sides. The alloy-coating powder was made from 65% copper, 18% nickel and 17% zinc.

Before virucidal testing, surfaces were cleaned using 70% ethanol followed by a soak in 70% ethanol for at least 5 min immediately before use. For repeated uses, immediately after virucidal tests concluded, surfaces were cleaned with 70% ethanol, followed by a warm deionized water rinse with light scrubbing. Before the repeated incubation, the surface was disinfected as before with a 70% ethanol soak.

### Surface tests

#### Wet virus droplet

For wet virus droplet tests, the experimental setup was based on bactericidal standard tests (JIS Z 2801, ISO 22196). As these tests specifically investigate bacteria, procedures were modified to test virucidal activity as follows. Coupons were placed in a 60 mm Petri dish and held at > 90% humidity in a sealed container. 750 µL of virus solution was pipetted onto the coupons, creating a pool on top of the coupon. A 10 µL sample was collected at various times and stored in 990 µL SF-900 III media (10^2^ dilution) at 4 °C until titering. All tests were performed in a biosafety cabinet in a temperature-controlled room set to 21 °C.

#### Dried virus droplet

For dried virus droplet tests, 25 µL of virus was dried onto the surface in a biosafety cabinet at ambient lab conditions (21 °C, ∼ 35%RH). Virus was resuspended in 25 µL of fresh media and 10 µL was collected and stored at 4 °C until titering.

#### Virus incubation with metal ions

In a 1.5 mL centrifuge tube, 700 µL of virus stock was incubated with 100 µL of each metal ion solution for a total volume of 1 mL. Conditions were tested in a 2^3^ factorial design experiment (Table 1). The high concentrations were Cu: 12.1 mM|Ni: 1.5 mM|Zn: 0.5 mM, and low concentrations were type-I ultra-pure water (UPW). Center points were half of the high concentration values and created using 50 µL of each metal ion solution and 150 µL UPW, for a total volume of 1 mL.

**Table 1:** Combinations of divalent metal cations in a factorial design of experiment (Fig. 7)

Combination	Copper	Zinc	Nickel	Log difference	
				Unfiltered	Filtered
Center point	**Mid**	**Mid**	**Mid**	− 2.62	− 3.76
A	*High*	*High*	*High*	− 3.57	− 4.72
B	***Low***	*High*	*High*	− 1.23	− 1.24
C	*High*	***Low***	*High*	− 3.26	− 4.79
D	***Low***	***Low***	*High*	− 0.52	− 1.23
E	*High*	*High*	***Low***	− 3.02	− 4.81
F	***Low***	*High*	***Low***	− 0.04	− 0.01
G	*High*	***Low***	***Low***	− 3.42	− 4.78
H	***Low***	***Low***	***Low***	0.00	− 0.01

#### Soiling agents

Two standard soiling agents were tested, Old EPA and New EPA. Old EPA was made by combining virus solution with FBS (5%, final concentration) and Triton X-100 (0.01%, final concentration). New EPA soiling solution was made of BSA (0.05 g/mL), yeast extract (0.05 g/mL), and bovine mucin (0.004 g/mL) in PBS. BSA and yeast extract solutions were filter sterilized (0.2 µm), while bovine mucin solution was autoclaved. A 1 mL soiled sample was made using stock solutions combined with the virus solution immediately before testing in the following ratios: 50 µL BSA stock, 70 µL yeast extract stock, 200 µL mucin stock, 680 µL virus solution.

### End-point dilution assay

Sf-9 cells from an exponentially growing suspension culture were diluted to 2.0 × 10^5^ cells/mL, and 100 µL of this dilution was seeded into each well of a flat-bottom tissue-culture treated 96-well plate (Fisher Scientific, Ontario, Canada). The cells were given at least one hour to attach, while kept inside of a humidified box at 27 °C. The virus was serially diluted in the range of 10^−2^ to 10^−8^, creating seven test dilutions. After the cells attached, 10 µL of the serial dilutions was added to each well in a column, creating 12 replicates in one plate. The diluent, media, was used as a negative control and added in 12 replicates. One 96-well plate was created for each experimental condition. Plates were incubated for seven days at 27 °C, after which they were checked for red fluorescence using a fluorescent microscope. Fluorescence was used as an indicator of infectious virus and titer was calculated using the method developed by Reed and Muench [17]. Results were reported as plaque forming units per mL (PFU/mL).

### Wet ashing

Virus samples were diluted (0.5 mL harvested virus sample in 9.5 mL UPW) and placed on a heating block set to 110 °C in a fume hood. 1 mL of aqua regia acid (3:1 molar ratio of hydrochloric acid to nitric acid) was slowly added and the mixture was allowed to reflux for 15 min. A further 2 mL of aqua regia was added slowly to the sample and refluxed for an additional 30 min. A digestion blank of 10 mL UPW was performed along-side the digestion to match the sample preparation. After wet-ashing was complete, samples were cooled to room temperature and filtered.

### Inductively coupled plasma optical emission spectroscopy (ICP-OES)

Samples were analyzed for 20 commonly occurring elements. Calibration curves with a range 0 to 100 ppm for each element allowed quantification. Samples were loaded in with an internal 10 ppm yttrium standard to adjust any changes in intensity cause by matrix effects using a Prodigy High Dispersion ICP (Teledyne Leeman Labs, New Hampshire, USA).

## Results

Virucidal activity by the copper–nickel–zinc alloy was tested in three main manners: wet virus droplet, dried virus droplet, and in solution with the alloy’s constituent metal ions. Each of these tests sought to elucidate different aspects in which the alloy surface causes inactivation and factors that may interfere in their testing. The wet virus droplet tests aimed to test virucidal activity using a current standard test, with a further investigation into the virus preparation methods that may interfere in those results. The dried virus droplet tests are more representative of hospital environmental conditions. Finally, the metal ion suspension tests sought insight into the virucidal activity of each major constituent metal for this alloy.

### Wet virus droplet tests

Virus droplet tests were performed at ≥ 90% relative humidity to prevent droplets from drying. These tests do not mimic realistic environmental conditions as virus will dry at the time-scale tested for the significantly lower relative humidity expected in hospitals. However, this method mirrors those outlined in current standard tests evaluating surface disinfectants (JIS Z 2801, ISO 22196) and follows the methods described in “Wet virus droplet” section.

#### Cell debris as a soiling agent

To create a testable virus stock, virus was amplified by infecting a host cell culture and resulted in an approximate tenfold increase in titer. When harvesting the amplified virus, cell viability was approximately 75% and density at 4 × 10^6^ cells/mL. These parameters were selected to minimize cell debris and virus degradation from the proteins released by lysed cells. Low speed centrifugation was used to remove cells and large debris (i.e., clarification). Smaller cellular debris typically remains and therefore the supernatant was filtered using a 0.4 µm and then a 0.22 µm filter.

To determine the effect of cellular debris as a soiling agent, a representative amount of debris was selectively generated and added to the filtered virus stock. Debris generated during the amplification in a cell-culture based setting was selected. Based on culture conditions, debris generated by 1 × 10^6^ lysed cells/mL is expected. Cells were grown to this density, collected using low speed centrifugation, resuspended in either fresh media or conditioned media, and sonicated to generate the simulated cellular debris soiling load. Conditioned media was taken from exponentially growing cells and low speed centrifugation was used for clarification. A further 1:10 dilution of the sonicated cells with the respective medias was also performed to generate a lower debris load. These solutions, along with fresh and conditioned media without debris, were used as diluents to investigate interference of the alloy’s virucidal effects by cell debris and growth media.

**Fig. 1:**
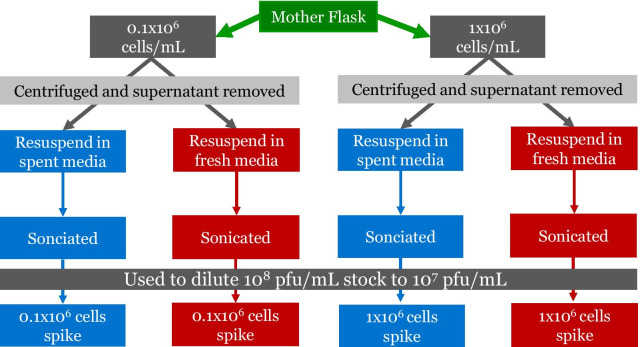
A diagram demonstrating the workflow for the experiment performed in Fig. 2A. Cells in the exponential growth phase (mother flask) were collected and resuspended in conditioned (spent) or fresh media. Conditioned media was made by clarifying media from the mother flask. These cell suspensions were then sonicated and used as the diluent to make the working virus solutions (10^7^ PFU/mL)

The tested conditions are outlined in the diagram in Fig. 1.

**Fig. 2:**
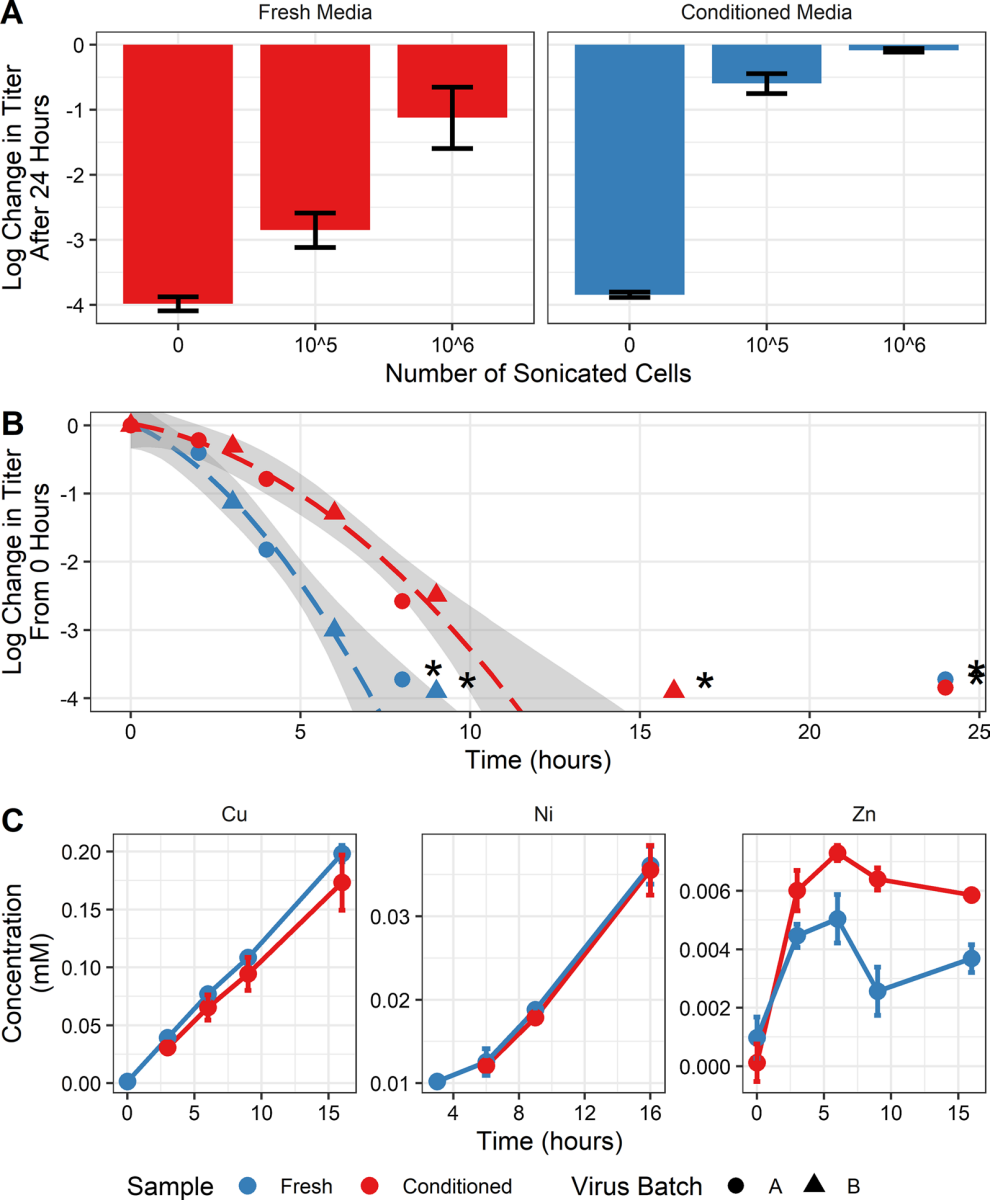
Inactivation of the virus on copper alloy was tested under various conditions. Virus was diluted into either fresh (red) or conditioned media (blue). Log difference represents the change from starting virus concentration to virus concentration at the specified time. Asterisks indicate data with at least one replicate at or below the detection limit. **A** Virus was exposed to the surface for 24 h with cell debris in the spike concentrations (x-axis). Each condition is the average of biological replicates (n = 2) with error bars representing the range of the data. **B** A time course analysis of virus inactivation in fresh or conditioned media only. Trend lines (dashed) with standard deviation (grey area) were calculated only using data above the detection limit. Virus Batch A (circles) is the geometric mean of two technical replicates and two biological replicates (n = 4). Virus Batch B (triangles) is the geometric mean of two technical replicates only (n = 2). **C** ICPOES of leachates from B up to 16 h. Only the alloy’s major constituents are presented. Each sample is the average of two technical replicates and two biological replicates (n = 4). Error bars represent the standard deviation. Lines are not the result of any regression but help to visualize trends in the data

Results presented in Fig. 2A are measured after a 24-h exposure to the copper alloy coated coupon. For conditioned media, addition of cells significantly prevented virucidal activity and was independent of the debris concentration (*p* < 0.001 for both). However, for fresh media, only the addition of 10^6^ sonicated cells was significantly different from no cells (*p* < 0.05). Comparing the media for the same level of cell debris showed that the inactivation in conditioned media was significantly lower than that of fresh media.

#### Conditioned media as a soiling agent

The protective effects of conditioned medium were investigated and is shown in Fig. 2B. Here, virus inactivation was investigated for up to 24 h. Virus was suspended in either conditioned or fresh media using two independently prepared batches of working virus stock.

In Fig. 2B, points at or below the detection limit (denoted by an asterisk) were not included in calculating the trend line. Model parameters were found to be significantly different between conditioned and fresh media (*p* < 0.05). These results indicate that virus in conditioned media followed a significantly slower inactivation (*p* < 0.05).

To determine if organic deposition from the soil was preventing ion leaching, ICP-OES was performed on these virus samples. These results are presented in Fig. 2C. Concentrations at each time point for copper, nickel, or zinc were not significantly different for soiling conditions (*p* < 0.05), indicating that soiling did not prevent leaching of metal ions.

#### Soiling agents used in standard tests

In developing a standard protocol for measuring antimicrobial activity of copper surfaces, the EPA initially proposed a soil made of PBS, FBS, and TX-100 (denoted Old EPA Soil). However, during public consultation of the protocol, it was suggested that the soil be changed to follow the current ASTM standard (denoted New EPA Soil) of FBS, yeast extract/yeastolate, and bovine/porcine mucin EPA [18]. Therefore, the effect of different soiling agents (conditioned media, Old EPA, and New EPA) was investigated.

**Fig. 3:**
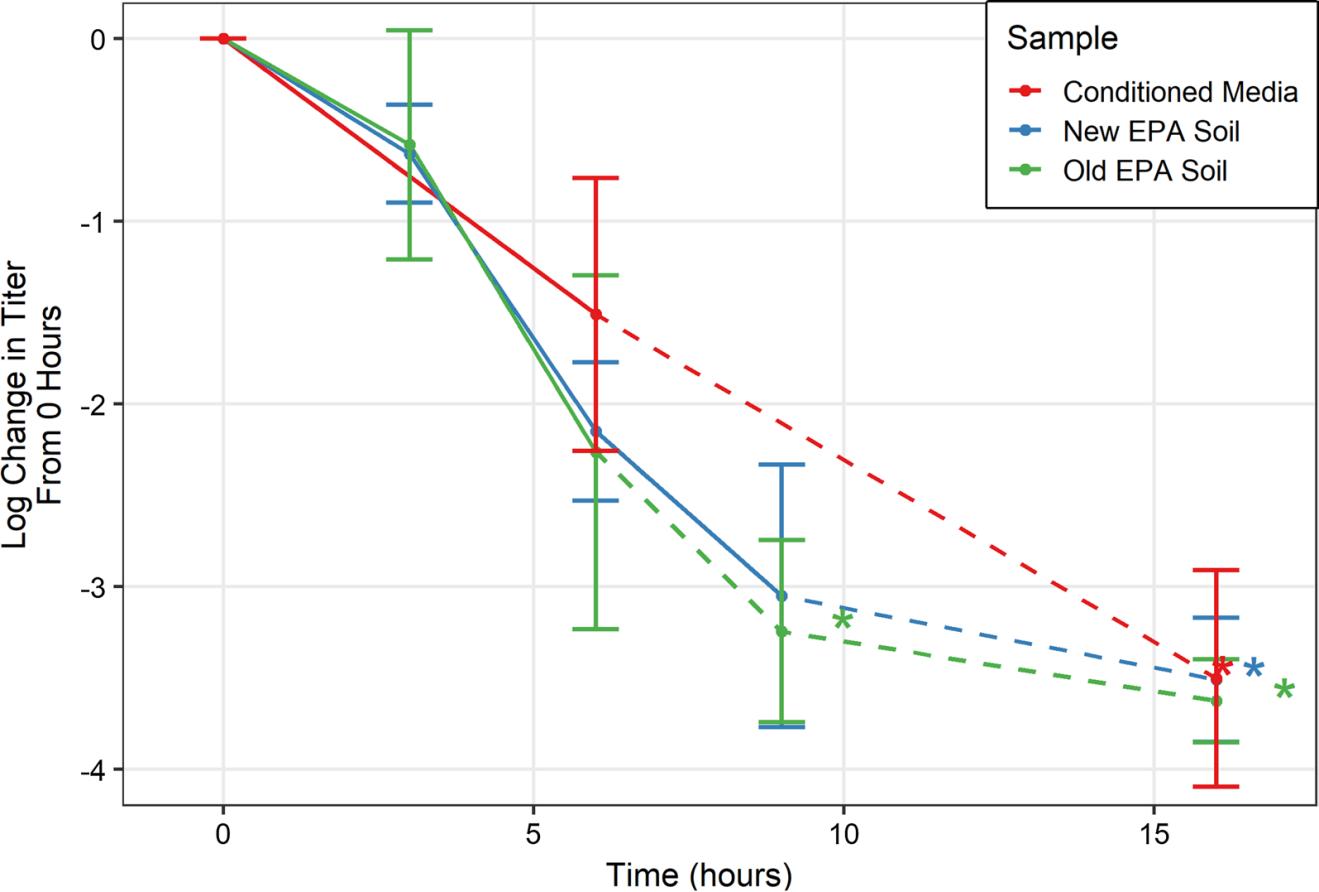
Titer of three different soiling conditions from 0 to 16 h. Lines are not the result of any regression but help to visualize trends in the data. Solid lines are between points in which all replicates were above the detection limit. Dashed lines indicate the next point had a replicate at or below the detection limit. Each point represents the geometric mean of two technical replicates and two biological replicates (n = 4). Error bars represent the standard deviation. Asterisks indicate at least one replicate was at or below the detection limit

No significant difference was found between any soiling condition at any time point (*p* > 0.05) as shown in Fig. 3. Solid lines connect the time points, while dashed lines indicate the next point was below the detection limit and are meant to help guide the reader’s eye. Points below the detection limit are indicated by an asterisk matching the sample colour.

#### Repeated surface exposures

This copper alloy surface is intended for a hospital setting where it is likely to become contaminated multiple times before being cleaned. A cursory investigation into the surface’s ability to repeatedly inactivate virus was performed. Further, a time series was used to discern any changes in inactivation kinetics.

**Fig. 4:**
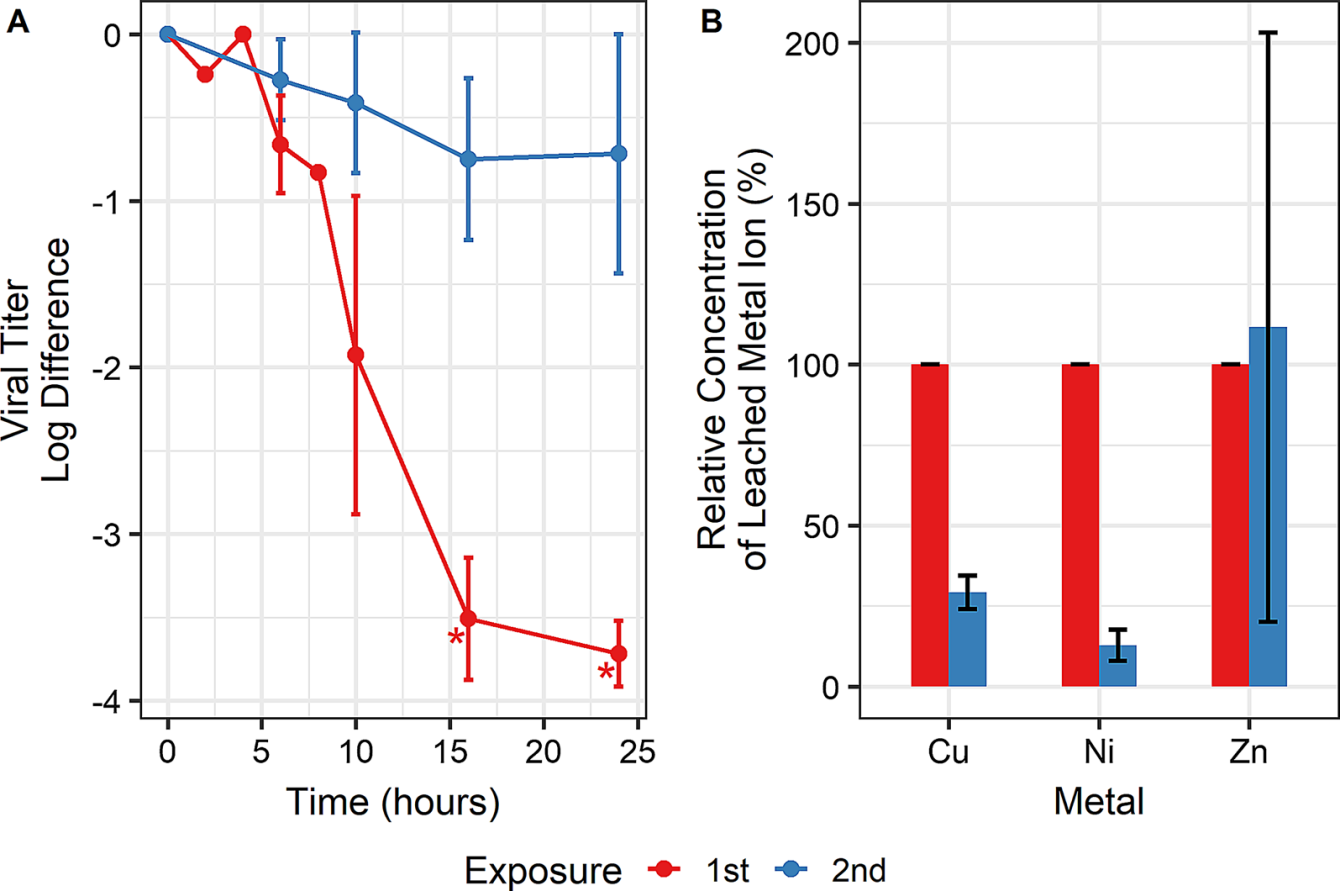
**A** Copper alloy was subject to two rounds of virus exposure. The first exposure is presented in red points and lines, and the second exposure is presented in blue points and lines. Each point represents the geometric mean of three biological replicates (n = 3). Error bars represent the standard deviation. Asterisks indicate at least one replicate was at or below the detection limit. Lines are not the result of any regression but help to visualize trends in the data. **B** ICP-OES of the samples collected for titering in **A**. Only the alloy’s major constituents are presented. Each bar represents the geometric mean of three biological replicates (n = 3) and are normalized to concentrations from the first exposure. Error bars represent the standard deviation

Using three biological replicates, it was found that the surface was unable to repeatably in-activate the virus. At 16 and 24 h, the second exposure coupons had a significantly reduced inactivation of virus (*p* < 0.05), as shown in Fig. 4A. Between exposures, the surface was cleaned using 70% ethanol and gentle scrubbing under running deionized water. Due to the drastic decrease in virucidal activity between the first and second exposure, a third exposure was not performed. ICP-OES of the repeated exposure showed that copper and nickel leaching decreased significantly (*p* < 0.05) on the second exposure (Fig. 4B).

### Dried virus droplet tests

In infected patients, expelled virus is typically in microliter volumes that dry fairly quickly in ambient humidity. The following tests evaluate the virucidal properties of the copper alloy surface using the methods described in “Dried virus droplet” section.

#### Inactivation of dried virus

**Fig. 5:**
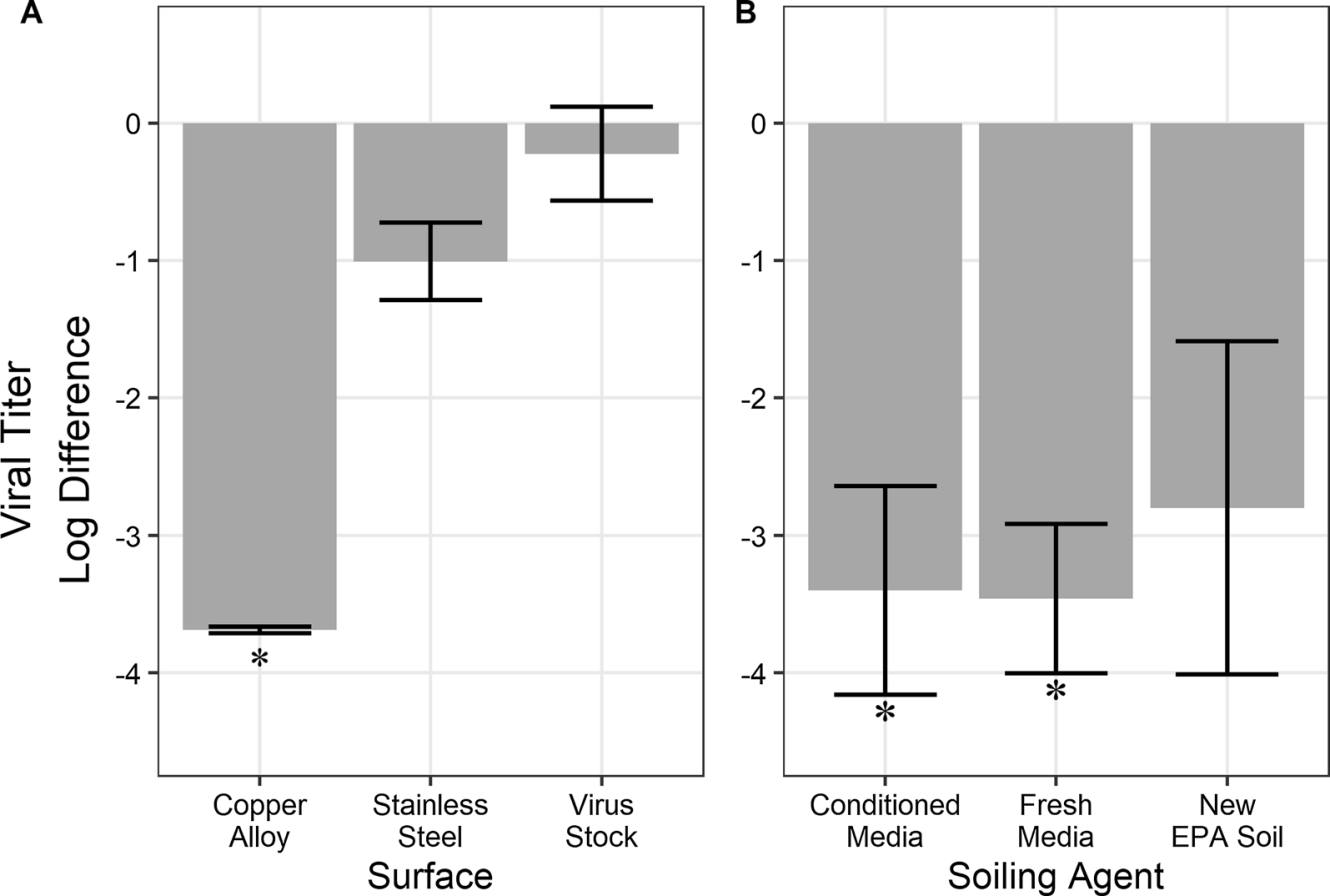
**A** Virus was dried onto either copper alloy or stainless steel. Virus stock represents virus exposed to only the ambient temperature and light in a sealed micro-centrifuge tube. Each bar is the geometric mean of two technical replicates and two biological replicates (n = 4). Error bars represent the standard deviation. Asterisk indicates at least one replicate was at or below the detection limit. **B** Virus suspended in three different matrices was dried onto the copper alloy. Each bar is the geometric mean of two technical replicates and two biological replicates (n = 4). Error bars represent the standard deviation. Asterisks indicate at least one replicate was at or below the detection limit

Inactivation of the dried virus was compared to stainless steel and the copper alloy. Drying of virus resulted in a 1-log loss (± 0.28), as shown by recovery from stainless steel (Fig. 5A). Virus was judged to have dried after 45 min in a biosafety cabinet using visual inspection. Copper alloy achieved inactivation beyond the detection limit (≥ 3.69-log) when in conditioned medium. Virus stock, a control for all ambient factors excluding humidity, did not have a significant change (0.22-log ± 0.34).

#### Soiling interference on dried virus inactivation

Virus was suspended in fresh media, conditioned media, or new EPA soil. No significant difference (*p* > 0.05) was found between conditioned media, fresh media, or New EPA soil and is presented in Fig. 5B. Virus in conditioned media and fresh media were inactivated beyond the detection limit (≥ 3.40-log ± 0.76 and ≥ 3.46-log ± 0.54, respectively), while the New EPA Soil samples still had detectable virus after drying (≥ 2.8-log ± 1.2).

#### Repeated surface exposures

Finally, the ability to repeatedly inactivate virus was tested. The same copper alloy surface was subjected to three virus exposures under the same soiling conditions. Between each exposure, the surface was cleaned by gentle scrubbing with 70% ethanol followed by a rinse with deionized water. Before reuse, the coupons were sterilized by immersing in 70% ethanol for at least 5 min.

**Fig. 6:**
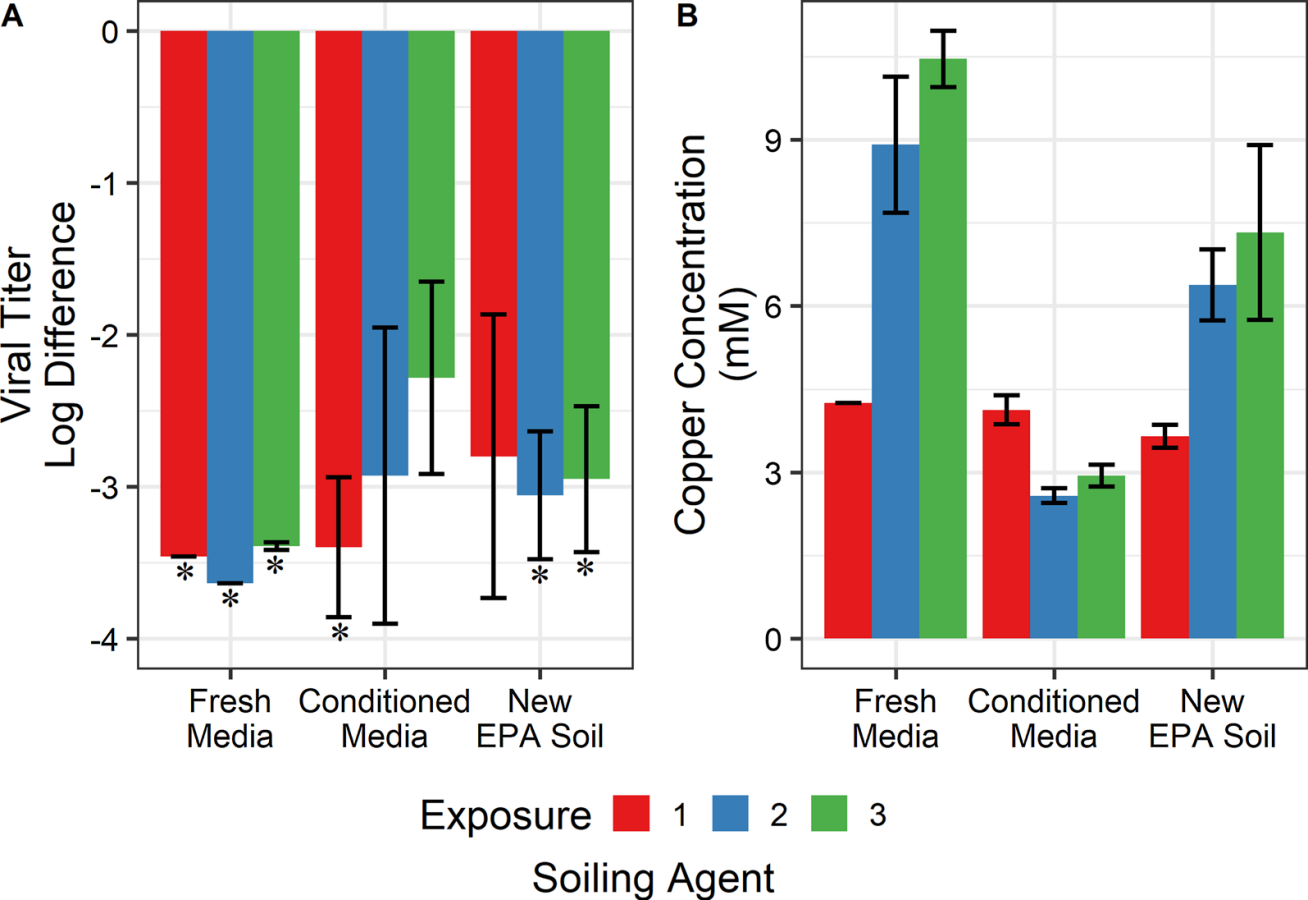
**A** As in Fig. 5B, virus suspended in three different matrices was dried onto the copper alloy. The alloy was exposed to each virus suspension a total of three times. Each bar is the geometric mean of two technical replicates and two biological replicates (n = 4). Error bars represent the standard deviation. Asterisks indicate at least one replicate was at or below the detection limit. **B** ICP-OES of samples collected in **A**. Only the copper ion concentrations are presented here for clarity. Error bars represent the standard deviation. Each colour corresponds to the exposure

No significant difference was found between each exposure or soiling condition (*p* > 0.05 for both), as shown in Fig. 6A. These results indicate that dried virus was continually inactivated by the surface.

For clarity, only the copper ions leached from the surface are presented in the ICP-OES data collected for these samples. The copper ion concentrations were also representative of alloy ions leached, as the ions leached in proportional concentrations.

### Metal ion solutions

A factorial experiment was performed using divalent cationic copper, nickel, and zinc based on leachate concentrations from the alloy surface. The high ion concentrations were those determined from a leachate (Cu: 12.1 mM|Ni: 1.5 mM|Zn: 0.5 mM), the center-points concentrations were half of the high level, and the low concentrations were UPW only. The virus sample was prepared in two different ways, as described in “Wet virus droplet tests” section (filtered) or by omitting the filtration through a 0.2 µm filter (unfiltered). As determined in Fig. 3, the unfiltered virus sample contains a significant amount of cell debris which may be sequestering metal ions, resulting in a lower virus inactivation.

**Fig. 7:**
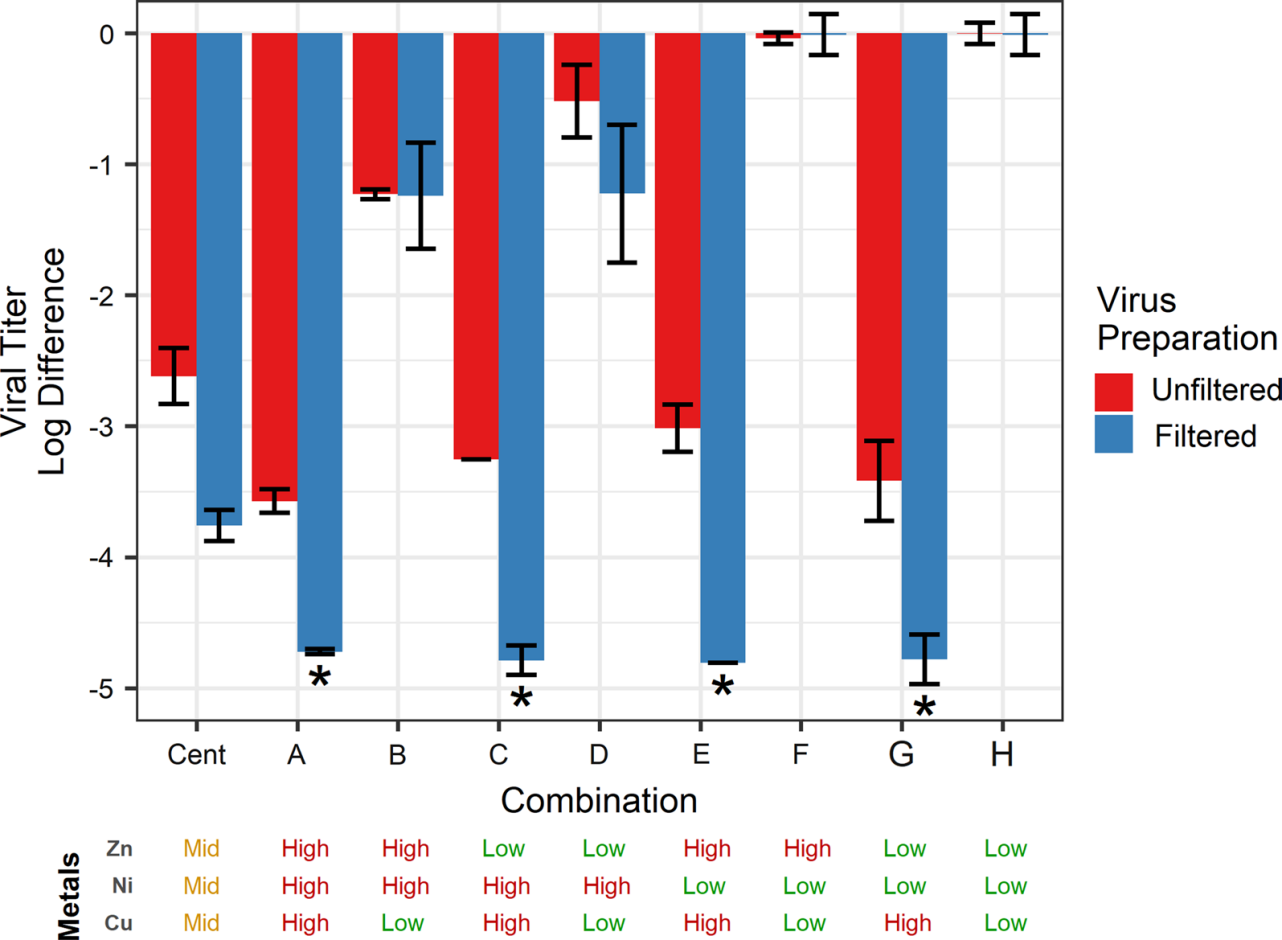
A factorial experiment testing the effect of copper, nickel, and zinc of virus inactivation in solution. Inactivation was tested using two different virus preparation procedures, unfiltered (red bars) and 0.2 µm filtered (blue bars). Combinations are as outlined in 1 and presented again below the figure. Each bar represents two technical replicates (n = 2), except in the case of center-points which had four technical replicates (n = 4). Asterisk indicates that at least one replicate was below the detection limit

Copper and nickel had a significant effect on reducing viral titer, and the interaction between copper and nickel was also significant for both methods of virus preparation as presented in Table 1 and Fig. 7. A significant difference was found between the preparation method for the centerpoint and combinations A, C, E, and G (Table 1). A further analysis on the unfiltered samples showed no significant difference between any of the copper combinations. Interestingly, the reduction by combination B (high zinc and nickel) was significantly greater than that of combination D (high nickel only). This difference indicates that there may be a synergistic effect between the two metals for virucidal activity.

## Discussion

The economic and physical burden of nosocomial infections have been well documented, with surface contamination identified as a significant source of these infections [1]. Several stringent methods have been developed to reduce the impact of surface contamination on nosocomial infection spread [1]. However, it appears that there are still significant issues with surface contamination persisting after thorough decontamination [4].

Research has shown both enveloped [19, 20] and nonenveloped [21] viruses can persist on high-touch non-porous surfaces for many days. Further, a review by Kutter et al. [22] found that the transmission routes for many enveloped viruses is complex but that fomites on hard surfaces were consistently implicated. To reduce surface-based transmission, several self-sterilizing technologies have been developed.

Copper surfaces have been developed and tested for antimicrobial activity [12], but there is a significant drop-off between lab-tested reductions and those seen in practice [23]. This shows that further standard method development is necessary, particularly in the preparation of tested viruses. Our goal was to first establish virucidal activity by a copper–nickel–zinc alloy and then explore potential sources of variability between lab and practical applications. This was achieved by investigating the virus matrix and variability in testing conditions.

When investigating virus droplet disinfection, our results showed that copper ion leaching was associated with the inactivation of virus. This has also been indicated in previous studies [21, 24]. We have also showed that soiling under wet conditions will significantly decrease the virucidal activity of copper as a self-sterilizing surface, indicating that virus sample preparation may bias the inactivation properties of the alloy. This is particularly important because many virucidal surface tests in literature utilize an unknown soiling level [21, 25].

Both cell debris and conditioned media acted as a soiling agent, but conditioned media appeared to have a much stronger effect than cell debris alone. Cell debris and conditioned media combined (representing high soiling) showed almost complete protection of the virus from any virucidal activity by the surface. It is likely that soil load is high in shedded infectious material, especially with viral infections, which require cell lysis as part of shedding.

Interestingly, the protective effects of soiling in wet conditions were not observed for dried virus. Since complete inactivation was achieved when the virus dried, developing a surface with faster drying time may be of interest. The inactivation of virus by copper alloys is consistent with literature data, as many of these studies were performed with small virus samples (< 50 µL) dried onto the surface [21, 26, 27].

To the authors’ knowledge, this was the first time concentrations of leached metals from a surface were directly compared to virucidal activity, as opposed to indirectly (using copper-chelating molecules such as EDTA) [27] or for bacteria [28, 29]. Crucially, we showed soiling did not prevent virucidal activity by inhibiting leaching of metals, but rather, likely by sequestering of metal ions. In our studies of soiling agents, both the current ASTM standard (New EPA Soil) and our conditioned media (undefined) had similar protective effects against virucidal activity of the surface. However, the authors recommend that the ASTM soiling be employed in all future studies, as it provides a uniform and consistent source of soiling across virus and host combinations.

This work on the virus matrix extended on tests of copper leaching by different solutions [28]. In their work, Molteni et al. [28] showed that a buffer known to dissolve copper (Tris–HCl) and conditioned medium were able to leach copper and completely kill *Enterococcus hirae* in 12 min. However, water and NaP_*i*_ buffer leached much lower copper concentrations and were not able to inactivate *E. hirae* for multiple hours [28]. In a more focused approach, we tested only fresh and conditioned media for the matrix and showed that the level of soiling will further alter the alloy’s virucidal properties.

Soiling has been recognized as an interfering factor for quite some time, and the use of standardized test protocols helps to normalize the variability from this factor [15]. Although there are no standard protocols in place for self-sterilizing virucidal surfaces, similar test conditions for disinfectants have good translatability (ie ASTM E2197 - 17e1, E1053 - 11). Only a few studies testing virucidal surfaces employed the soil loads specified in those standards. To shed some light on accurate soil loads, it would be beneficial to look at organic soil loads in virus samples, possibly by quantifying total protein, lipid, and nucleic acid content. Therefore, it is worth investigating these interfering factors to ameliorate the difference between lab and field tests.

This study did not investigate the effects of humidity as it has been shown that humidity does not impact the efficacy of copper’s virucidal activity [13]. Additionally, a study investigating the effect of humidity on Influenza A stability found that relative humidity had no significant impact on survivability [30]. Since Influenza A is an enveloped virus that is fairly prone to environmental stresses, it can be reasonably assumed that relative humidity will only impact the drying time for many viruses. However, if the evaporation rate of the virus is linked to the efficacy of the disinfectant (as with ethanol [31] or silver surfaces [13]), drying should be controlled to achieve a roughly standard drying time. When using ‘ambient’ humidity, it should still be recorded as the levels can change, with seasonal fluctuations ranging from 15 to 48% in one study [32]. To this end, it would not only be beneficial to monitor the temperature and humidity in the lab, but also the tested clinical settings to help delineate ambient condition effects from other environmental factors in the clinical tests.

Some limitations of this study were the viral assay method and virus selection. Our experimental set-up prevented us from being able to detect complete inactivation of virus, with a detection limit of 2.13 × 10^3^ PFU/mL. Additionally, the tested virus is not of clinical relevance. However, baculovirus is stable for many environmental conditions [33] and may be considered a conservative surrogate for other enveloped viruses. Further, ease of genetic manipulation allowed easier quantification, and safe handling made baculovirus a good candidate for method development. In only testing an enveloped virus, we considered a scenario in which some soiling has occurred, but with a virus that was susceptible to environmental stresses. Non-enveloped viruses are less susceptible to environmental factors, although there are data to suggest that they will also be inactivated by copper ions [27].

In conclusion, a copper–nickel–zinc alloy was shown to have strong virucidal effects for a presumed conservative enveloped virus surrogate. Copper, nickel, and zinc ions were all shown to leach from the alloy surface and are the likely cause of virucidal activity by this surface. Virucidal activity was achieved under moderate soiling, but lost under high soiling generated by routine virus amplification procedures. The surface was able to repeatably inactivate dried virus droplets under moderate soiling conditions, but unable to do so for virus droplets kept wet using high humidity.

## Data Availability

All data and material are available upon request.
